# FOSS-Based Method for Thin-Walled Structure Deformation Perception and Shape Reconstruction

**DOI:** 10.3390/mi14040794

**Published:** 2023-03-31

**Authors:** Huifeng Wu, Rui Dong, Qiwei Xu, Zheng Liu, Lei Liang

**Affiliations:** 1 School of Electronic Information and Automation, Guilin University of Aerospace Technology, Guilin 541004, China; huizifeng_829@163.com (H.W.);; 2 National Engineering Research Center of Fiber Optic Sensing Technology and Networks, Wuhan University of Technology, Wuhan 430070, China; 3 Engineering Comprehensive Training Center, Guilin University of Aerospace Technology, Guilin 541004, China

**Keywords:** BP neural network, one-class SVM, fiber-optic sensor system, shape reconfiguration

## Abstract

To improve the accuracy of deformation perception and shape reconstruction of flexible thin-walled structures, this paper proposes a method based on the combination of FOSS (fiber optic sensor system) and machine learning. In this method, the sample collection of strain measurement and deformation change at each measuring point of the flexible thin-walled structure was completed by ANSYS finite element analysis. The outliers were removed by the OCSVM (one-class support vector machine) model, and the unique mapping relationship between the strain value and the deformation variables (three directions of x-, y-, and z-axis) at each point was completed by a neural-network model. The test results show that the maximum error of the measuring point in the direction of the three coordinate axes: the x-axis is 2.01%, the y-axis is 29.49%, and the z-axis is 15.52%. The error of the coordinates in the y and z directions was large, and the deformation variables were small, the reconstructed shape had good consistency with the deformation state of the specimen under the existing test environment. This method provides a new idea with high accuracy for real-time monitoring and shape reconstruction of flexible thin-walled structures such as wings, helicopter blades, and solar panels.

## 1. Introduction

Due to technical or economic factors, some special flexible thin-walled structures, such as helicopter blades, aircraft wings, wind-turbine blades, solar panels, etc., are difficult to achieve real-time structural deformation perception. However, due to the continuous development of technology and the attention of scholars, the methods and means of monitoring the deformation of these flexible thin-walled structures have been diversified and matured in recent years. The method is based on the image As [1,2,3,4,5], inverse finite-element method (iFEM) [6,7,8,9,10,11,12], and fiber-optic sensing method [13,14,15,16,17,18,19,20,21]. All the above methods have advantages and disadvantages. The iFEM method is based on the strain–displacement relationship, and the no-force balance, especially in setting boundary conditions, which makes the iFEM model extremely limited. The method based on the image has the advantage of image HD, though it needs to acquire many high-definition images, which means the method needs to obtain a large amount of data, which is an obstacle in the real-time process. Furthermore, the method will need precise installed position and high accuracy of the angle of the installation. In the optical fiber sensing method, optical fiber grating is light in weight and small in volume, so it is easy to form curvature (shape) sensors of various structures. The disadvantage is too dependent on the curvature (shape) of the sensor structure, and the calibration errors that occur continuously accumulate.

Machine learning has become one of the most efficient tools for complex problems through the promotion of big data [22,23,24,25,26,27,28,29]. The organic integration of data-driven and optical fiber-sensing technology alleviates the constraints brought by the economy and the technology and also minimizes the impacts of unknown factors to a certain extent. A neural network is one of the more widely used machine learning methods, and neural networks are more widely used in deformation perception; for example, the predicting of slope displacements with neural networks [30,31] and predicting widespread stomach deformation with neural networks [32]. Sefati, S et al. used sensory data from unmodeled uncalibrated sensors embedded in a continuum manipulator (CM) to estimate the shape and distal-end position estimation (DPE), studied three data-driven models, and compared DPE and shape-reconstruction results. The proposed neural network approach avoids the accumulation of integration errors in traditional methods and produced more accurate DPE [33]. Zheng, HR et al. demonstrated a kind of optical fiber shape sensing algorithm based on an artificial neural network, which provides improved accuracy, has better robustness, and has less time delay. The algorithm shows great potential for applications in high-precision real-time fiber-shape measurement [34]. Manavi, S et al. trained a neural network with supervised deep learning to extract shape information directly from Edge-FBG spectra and predicted the shape of a fiber optic sensor consisting of five edge-FBG triads by the network model. The tip error is less than 6 mm [35]. Baldwin, CS et al. used the strain of the structure to determine the deformation of the wing structure (cantilever plate). The experiment proved that the neural network approach performed best when trained using experimentally measured strain and deformation data [36]. Wu, XY et al. proposed a PSO-RR (particle swarm optimization-ridge regression) algorithm for structural deformation monitoring and reconstruction of a wing under different loading conditions. Compared with the KO theoretical results, the method is highly accurate and does not depend on the specific structure [37]. Wu, HF et al. proposed a method based on FBG and neural networks for flexible-structure deformation monitoring and shape reconstruction. The experimental results showed that the prediction results were highly consistent with the expected effect [38]. The above research shows the advantages of neural networks for deformation monitoring, especially the combination of neural networks and finite elements [39]. Neural networks also have many advantages for monitoring the deformation of thin-walled plates, though, in this area of application, the literature is not common at present.

To the existing problems of flexible thin-walled structure deformation perception, such as strong dependence on experience, mathematical models, and structure-specific curvature (shape) sensors, this paper proposes a method based on the combination of FOSS (fiber optic sensor system) and data drive, using the strain information measured by FOSS laid on the flexible thin-walled structure to complete the deformation perception and shape reconstruction. Based on the team’s previous research (The research results the authors collated for publication in *Micromachines* [38], this paper improves on three aspects:

(1) For the problem of sensor arrangement in thin-walled structures, the idea of using double FBGs for each measuring point is proposed to improve the accuracy of deformation perception of thin-walled structures, and the influence of sensor placement on the accuracy of deformation prediction is quantitatively analyzed by using the Ansys finite-element model;
(2) For the problem of outliers in the measurement process, the OCSVM (one-class support vector machine) model is used, which not only effectively eliminates the outliers and ensures the accuracy of shape reconstruction, but also provides a basis for judging abnormal conditions such as structural damage;
(3) Aiming at the shape-reconstruction error of thin-walled structures, analyze the prediction error of the neural network model, and the calculation error of the interpolation method for the shape-reconstruction error of thin-walled structures in this paper, and provide a reliable basis for improving the accuracy of the shape-reconstruction method.

## 2. FOSS and System Simulation

### 2.1. FBG Sensor Layout

**Figure 1:**
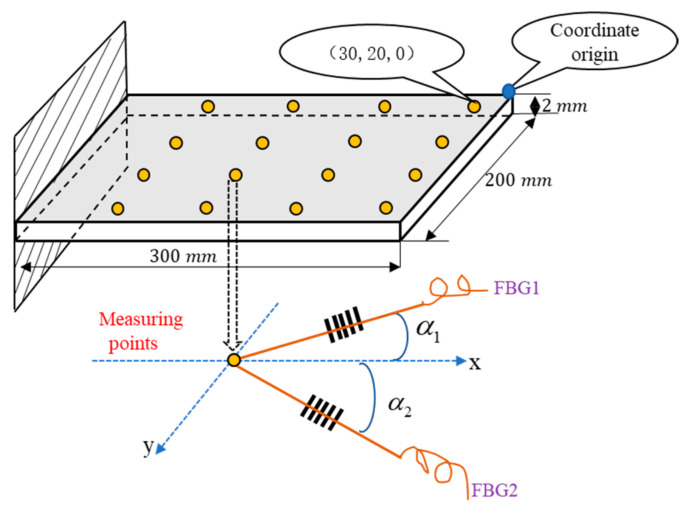
Structural drawing of a test piece with a flexible thin-walled structure.

In this paper, the authors are talking about flexible thin-walled structures, mainly similar to wind-turbine blades, solar panels, aircraft wings, helicopter blades, other flexible structures, and so on. That is, one end is fixed and the other end moves freely. The stainless-steel plate of 300 mm (length) × 200 mm (width) × 2 mm (height) is selected as the test piece for the flexible thin-walled structure in this system, as shown in Figure 1. The purpose is to verify the use of a data-driven mode for deformation sensing, which can simplify the impact of measurement due to uncertainties such as irregular shape and complex forces.

The layout of the FBG sensor can follow the optimization algorithm, such as the particle-swarm algorithm (PSO), considering the small area of the test piece and the force distribution in Figure 1, the system adopts the uniform distribution method to tile measuring points, a total of 16 measuring points, which is a combination of 4 rows ×4 columns. The distance of each row is 50 mm, the distance of each column is also 50 mm, the long side of the test piece is the x-axis, the width of the y-axis, the blue dot is the coordinate origin, and the coordinate of the nearest grating measuring point to the coordinate origin is (30, 20, 0). 

Combining the characteristics of the loads (vertical forces and torques) applied to the structure, inspired by the dual FBG shape sensor designed in the literature [40], one measuring point has two gratings, as shown in Figure 1, the angles between the FBGs of the measuring point and the x-axis are $\alpha_{1}$ and $\alpha_{2}$, respectively, and angles ($\alpha_{1}$, $\alpha_{2}$) will be analyzed in the next section.

### 2.2. Simulation of Deformation Perception System Based on FOSS

**Figure 2:**
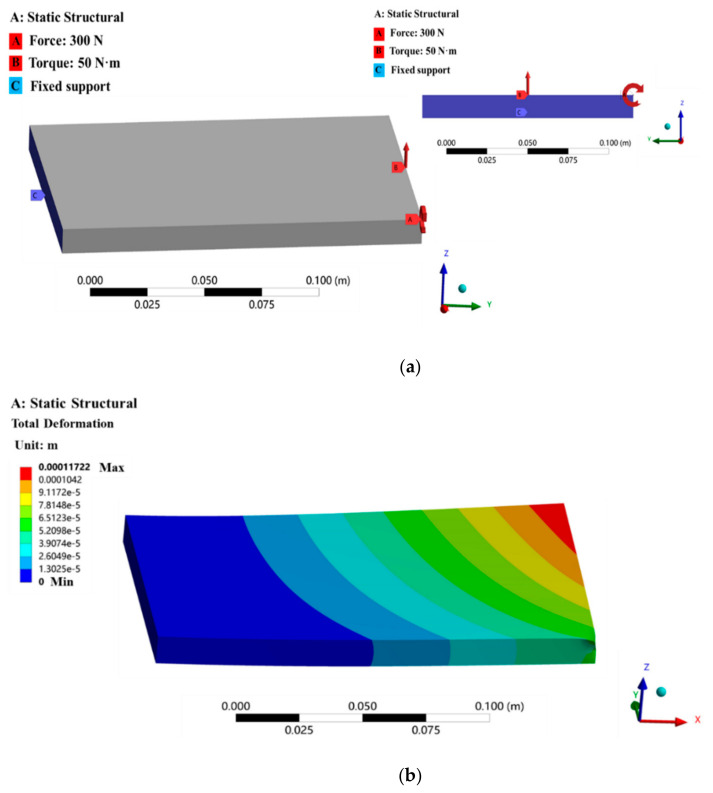
Simulation model of FBG perception system: (**a**) Model loading state diagram; (**b**) Model deformation cloud map.

For the structure of the test piece shown in Figure 1, the system simulation model shown in Figure 2a is established by the workbench module of the Ansys finite element software version 2022 to simulate the strain values and deformation variables of each measuring point on the test piece under different loads. The parametric method in the workbench is used to simulate the deformation and strain of the test piece under different loads. The input parameters are the value and the loading point of the load on the test piece, while the output parameters are the strain values and deformation variables in the x, y, and z directions of 16 measuring points on the test piece.

The torque is 50 Nm, the coordinate of the action point is (1.0), the force is 300 N, the coordinate of the action point is (0.100), and the cloud diagram of the test piece deformation is shown in Figure 2b.

When the force is positive, it means the direction of force is vertical upward, otherwise the opposite, torque is negative clockwise, positive counterclockwise.

For flexible thin-walled structures, differences in the value and the loading point of the load can lead to differences in the degree of structural deformation. The load is loaded on the test piece in six cases, as shown in Table 1: (Fx Fy—coordinates of the loading point of the force, Tx Ty—coordinates of the loading point of the torque).

The angular variations of the two gratings are shown in Table 2, and there are 10 combinations of angles.

There are four rows of 16 measuring points on the test piece, and one measuring point in each row is randomly selected, compared with the strains measured by the FBG for different angles between the FBG and the x-axis, under the loading of the six other loads listed in Table 1. Randomly selected measuring points of 1, 7, 11, and 16 and the strains of two FBGs at 10 different angles for each measuring point are shown below.

**Figure 3:**
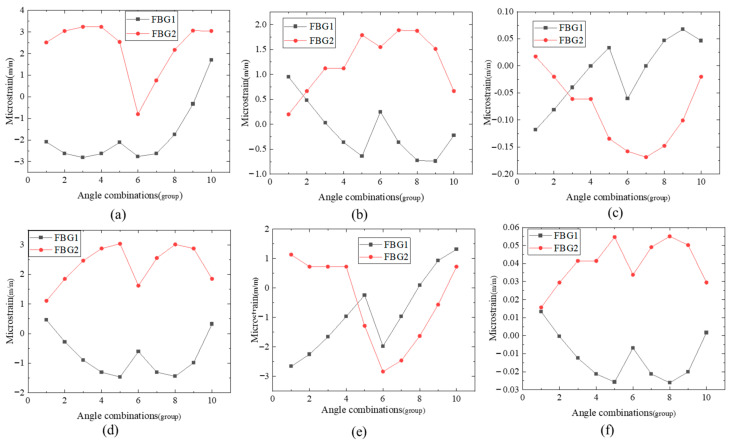
Double FBG strain variation diagram of measuring point 1: (**a**) Load loading mode 1; (**b**) Load loading mode 2; (**c**) Load loading mode 3; (**d**) Load loading mode 4; (**e**) Load loading mode 5; (**f**) Load loading mode 6.

**Figure 4:**
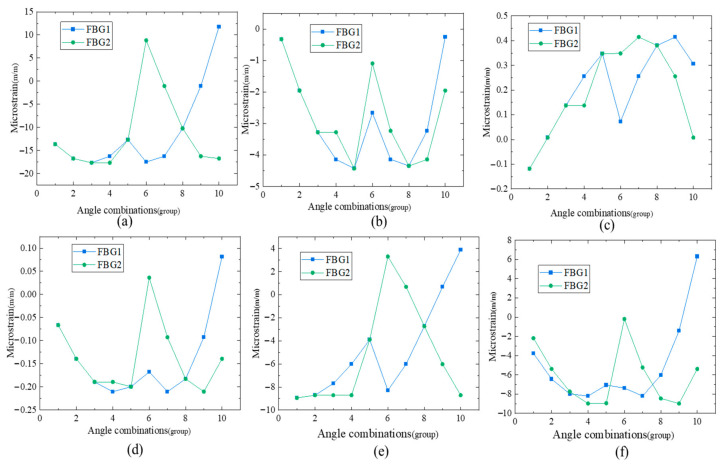
Double FBG strain variation diagram of measuring point 7: (**a**) Load loading mode 1; (**b**) Load loading mode 2; (**c**) Load loading mode 3; (**d**) Load loading mode 4; (**e**) Load loading mode 5; (**f**) Load loading mode 6.

**Figure 5:**
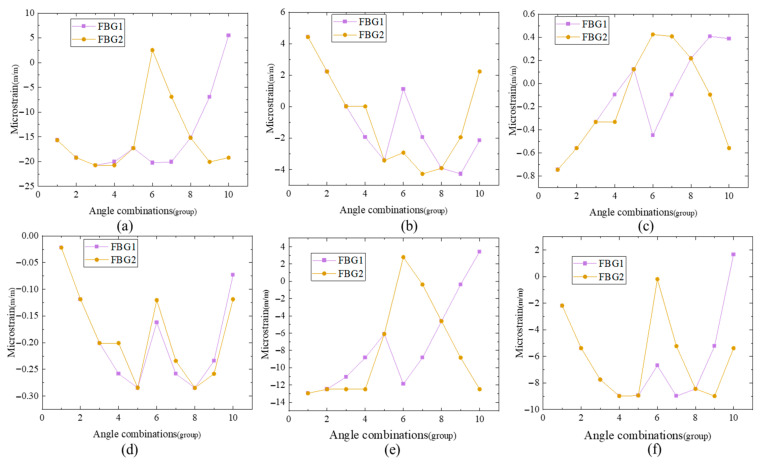
Double FBG strain variation diagram of measuring point 11: (**a**) Load loading mode 1; (**b**) Load loading mode 2; (**c**) Load loading mode 3; (**d**) Load loading mode 4; (**e**) Load loading mode 5; (**f**) Load loading mode 6.

**Figure 6:**
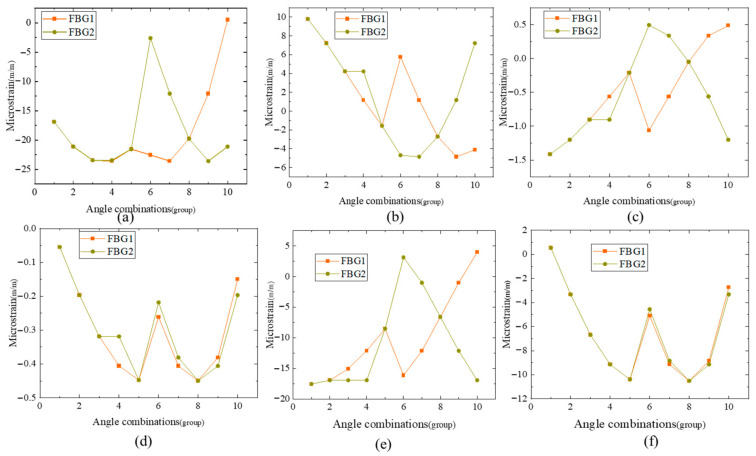
Double FBG strain variation diagram of measuring point 16: (**a**) Load loading mode 1; (**b**) Load loading mode 2; (**c**) Load loading mode 3; (**d**) Load loading mode 4; (**e**) Load loading mode 5; (**f**) Load loading mode 6.

As can be seen from Figure 3, Figure 4, Figure 5 and Figure 6, the strain values measured by the FBGs for each measuring point are different when the angle combination is not the same under the same load. To select the most suitable angle combination, the angle combinations are sorted by quantitatively analyzing the strain values measured of FBG at each measuring point. For example, for measuring point one, when the torque acts alone, the strain values of combinations one, six, and ten are smaller in comparison, while combinations five and eight have larger strain values, followed by combinations four and nine; when the force acts alone, combinations one and ten have larger strain values, followed by combinations two and nine, with the worse performance being combination six; when the force and torque act simultaneously, a comprehensive comparison of combinations five, eight, and nine have a better sensitivity to strain. 

Considering the combination, combination nine (the angle between the two FBGs and the x-axis is ±45°) is selected as the angle between the FBGs of the measuring points.

## 3. Machine Learning Methods

### 3.1. Data Preparation

In the simulation system shown in Figure 2, 6000 data samples are collected by the Ansys finite element model. To establish the mapping between the strain values and the deformation variables of the measuring point, the strain values (FBG1, FBG2) of the measuring points are selected as the feature, and the corresponding deformation variables (x-, y-, and z-axis) as the label to establish the sample data set of the neural network. 

### 3.2. Data Preprocessing

Using Ansys finite element method to collect sample data, when setting simulation boundary conditions, the system defaults that all data are within the range of elastic deformation, however, there are still samples of inelastic deformation, which is called a negative sample. Before the model training, these negative samples need to be eliminated, which may be inelastic deformation. The method of kicking out these negative samples is one class of SVM (support vector machine) in machine learning. 

OCSVM (one-class support vector machine) algorithm, strictly speaking, is not an outlier detection algorithm, but a novelty detection algorithm, that is, OCSVM starts from normal data and regards all the data different from normal data as novel data, and then sets the boundary according to the actual needs, and the data beyond the boundary can be judged as abnormal data. 

The characteristics of the deformation variables in the three axes of the measuring point are obvious, and the deformation variable in the z-axis is the largest, which is 104 times that of the x-axis and y-axis. To reduce the amount of calculation, instead of choosing the dimensionality reduction, the authors choose the simplest way—two feature quantities as one classification input feature. In the first row of measuring points with relatively large deformation variables, the authors randomly selected two deformation variables in the z-axis as input features since there was no sample with the same deformation variable in the z-axis in all 6000 samples. In the Matlab software version 2021, after installing the LIBSVM library, select reasonable performance parameters to build the OCSVM model by SVM toolbox in Matlab 1.0 of LU Zhen-bo.

**Figure 7:**
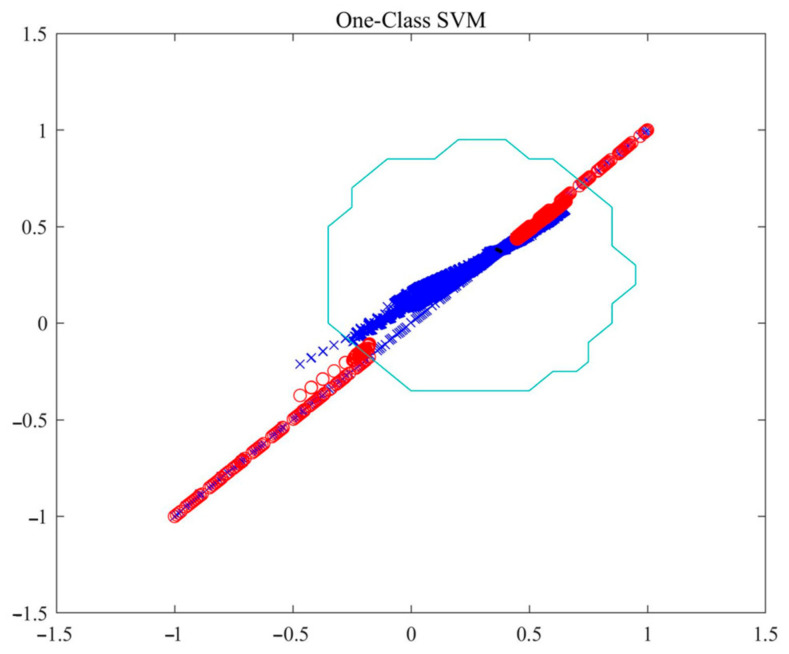
Classification diagram of outliers in the OCSVM model.

Select reasonable performance parameters to build the OCSVM model, ocsvm = OneClassSVM (kernel = ‘linear’, nu = 0.1), the kernel function of the model is the linear kernel. The test output is shown in Figure 7.

As can be seen from the final results, 605 samples are kicked out of 6000 samples, with a missing report rate of 0.39% and a false report rate of 5.75%. When creating the model, there were about 10% abnormal data in the default data of setting parameters nu = 0.1, and the performance of the established OCSVM model on clean data achieved the desired results.

### 3.3. Establishment of the BP Neural Network Model

The neural network is one of the common mathematical models in machine learning. In theory, the neural network can fit any function. In the training of sample data, the weights and thresholds of the network are constantly adjusted by backpropagation, and the gradient descent method is used to reduce the network error function. Finally, the predicted value approaches the expected output value.

#### 3.3.1. Model Selection

Due to the deformation variables in the z-axis differing from those in the x- and y-axes by 10^4^ orders of magnitude, in this system, a BP neural network is used to establish two sets of mapping relations, between the strain value and the deformation variables in the z-axis of the measuring point, and between the strain value and the deformation variables in the x-and y-axis of measuring point, respectively. The mapping relationship between the x- and the y-axis is used as an example to illustrate. 

**Figure 8:**
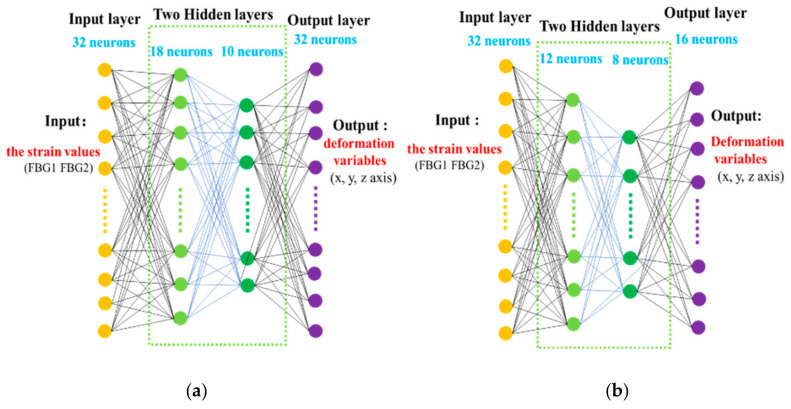
Schematic of the neural network model: (**a**) Model of the x- and y-axis; (**b**) Model of the z-axis.

After repeated experiments and continuous adjustment, the structure of the neural network is shown in Figure 8.

#### 3.3.2. Hyperparameter Setting

The learning rate of the network is 0.01 (the x- and y-axis), The learning rate of the network is 0.0001 (the z-axis), the maximum number of training is 10^4^, the target error to be achieved by the training network is 10^−6^, and the activation function is chosen as the sigmoid function, as shown in Equation (1).

(1) $$\delta \left(x\right)=\frac{1}{1+e^{-x}}$$

The distribution of the sample data is random, for convenience of calculation, out of the 5395 samples left after eliminating outliers, 5000 of them are arbitrarily selected, of which 75% of the samples are used for training, 15% for validation, and 15% for testing.

#### 3.3.3. Model Evaluation

To evaluate the predictive performance of the model, the residual of the predicted value, determination coefficient (R2), MSE (the mean squared error), and so on, are considered comprehensively.

**Figure 9:**
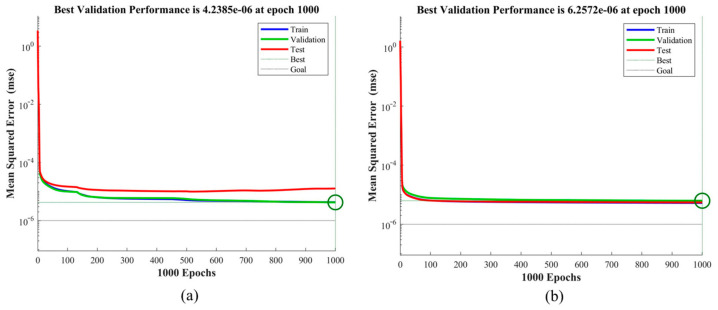
Network performance change diagram of model training process: (**a**) MSE of the x- and y-axis; (**b**) MSE of the z-axis.

**Figure 10:**
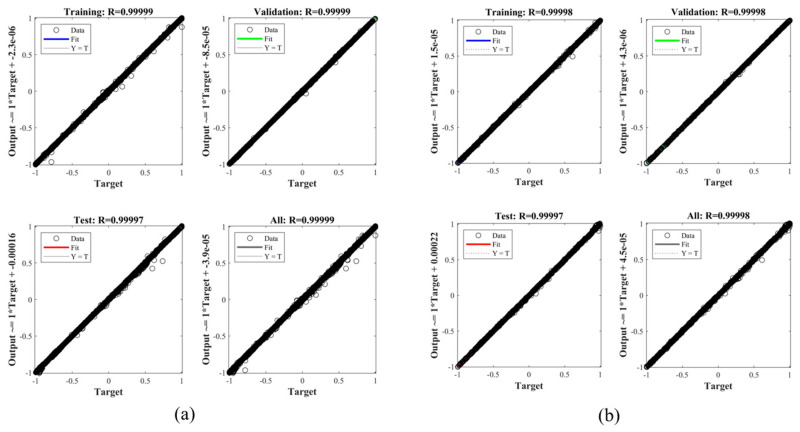
Correlation analysis of the model: (**a**) R2 of the x- and y-axis; (**b**) R2 of the z-axis.

MSE and R2 are important indicators for evaluating the prediction performance of neural network models. The closer the MSE is to zero, the better the model selection and fitting effect, and the more successful the data prediction. The closer R2 is to one, the better the model fit is. From Figure 9 and Figure 10, it can be seen that when the system builds the neural network model, the MSE eventually converges to zero and the R2 takes values close to one, which reflects the good prediction performance of the system.

**Figure 11:**
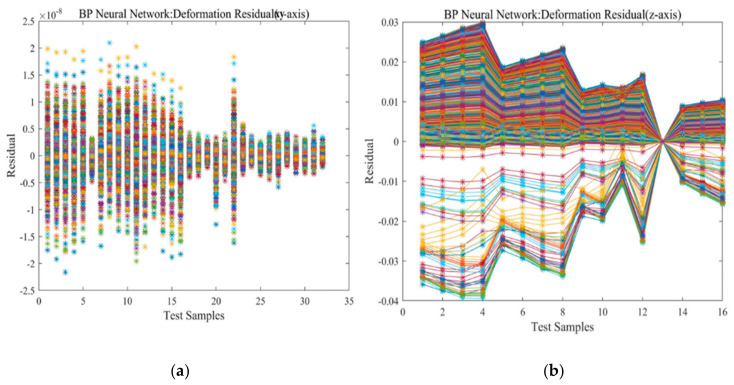
Residuals of the mode: (**a**) the x- and y-axis; (**b**) the z-axis.

The model residual is the difference between the predicted value and the actual value. From Figure 11a, it can be found that the residuals of measuring point four and measuring point eight are the largest in the x- and y-axis, though the maximum residual is <2.5 × 10^−8^ m, which is within the acceptable range; in Figure 11b, the residual of measuring point 13 is almost zero, which means that the predicted value of measuring point 13 is the most accurate, and the residual of measuring point four is larger, with a value range of (−0.04 m–0.03 m), which is also within the acceptable range.

### 3.4. Prediction of New Samples

To test the accuracy of the predicted values of the neural network model, the simulation model, as shown in Figure 2, produced a set of strain values after being loaded, and formed the variables of the measured points. The loading information is shown in Table 3 (x.y represents the coordinates of the loads).

The predicted values of the neural network model and the theoretical values of the system simulation for any 4 of the 16 measuring points on the test piece are shown in Table 4.

The flexible thin-walled structure will deform by loading, and the degree of deformation is directly related to the size and type of the load. By observing the characteristics of the theoretical and predicted data in Table 4, it finds that the structure has a large deformation in the z-direction (up and down in the direction perpendicular to the structure), in the x and y directions, the deformation is complex (the structure swings or rotates along the axial direction), and in general, the angle of swings or rotations of the flexible thin-walled structures is not too large within the elastic deformation range. As shown in Table 4, the deformation variables in the x-axis and y-axis are all μm levels, and the y-axis is even smaller. The error percentage of the predicted model in the x-axis and y-axis is relatively large, which has a small impact on the actual shape reconstruction and can be ignored due to its small numerical value, while the deformation variable in the z-axis is large, the maximum percentage error of the predicted model is 12.22%, and the larger the deformation variables of the measuring point are, the smaller the percentage error is, which effect on the shape reconstruction is limited.

In comprehensive comparison, there are errors in the prediction of the model in each direction, the influence of the error in the x-axis and y-axis on deformation reconstruction can be ignored, and the error in the z-axis has an impact on the shape reconstruction, though within an acceptable range, so the structure deformation perception method based on neural network is feasible.

## 4. Experiments and Results

**Figure 12:**
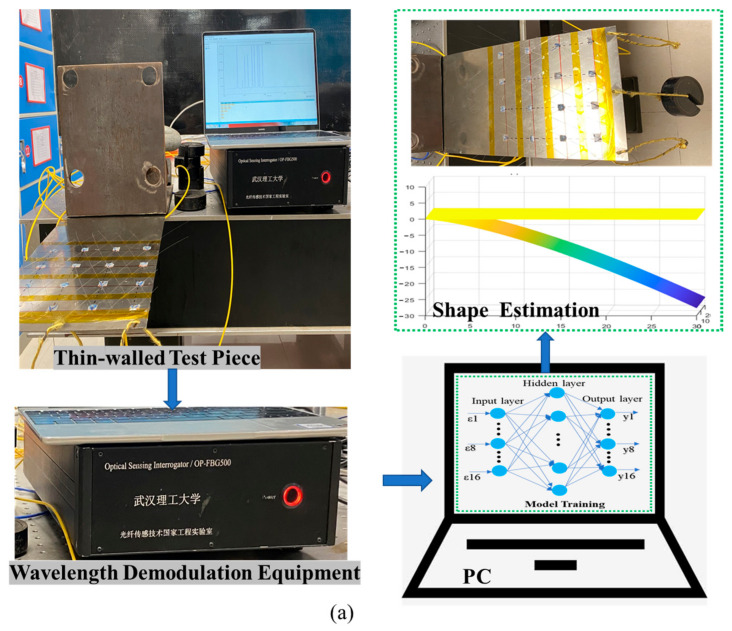

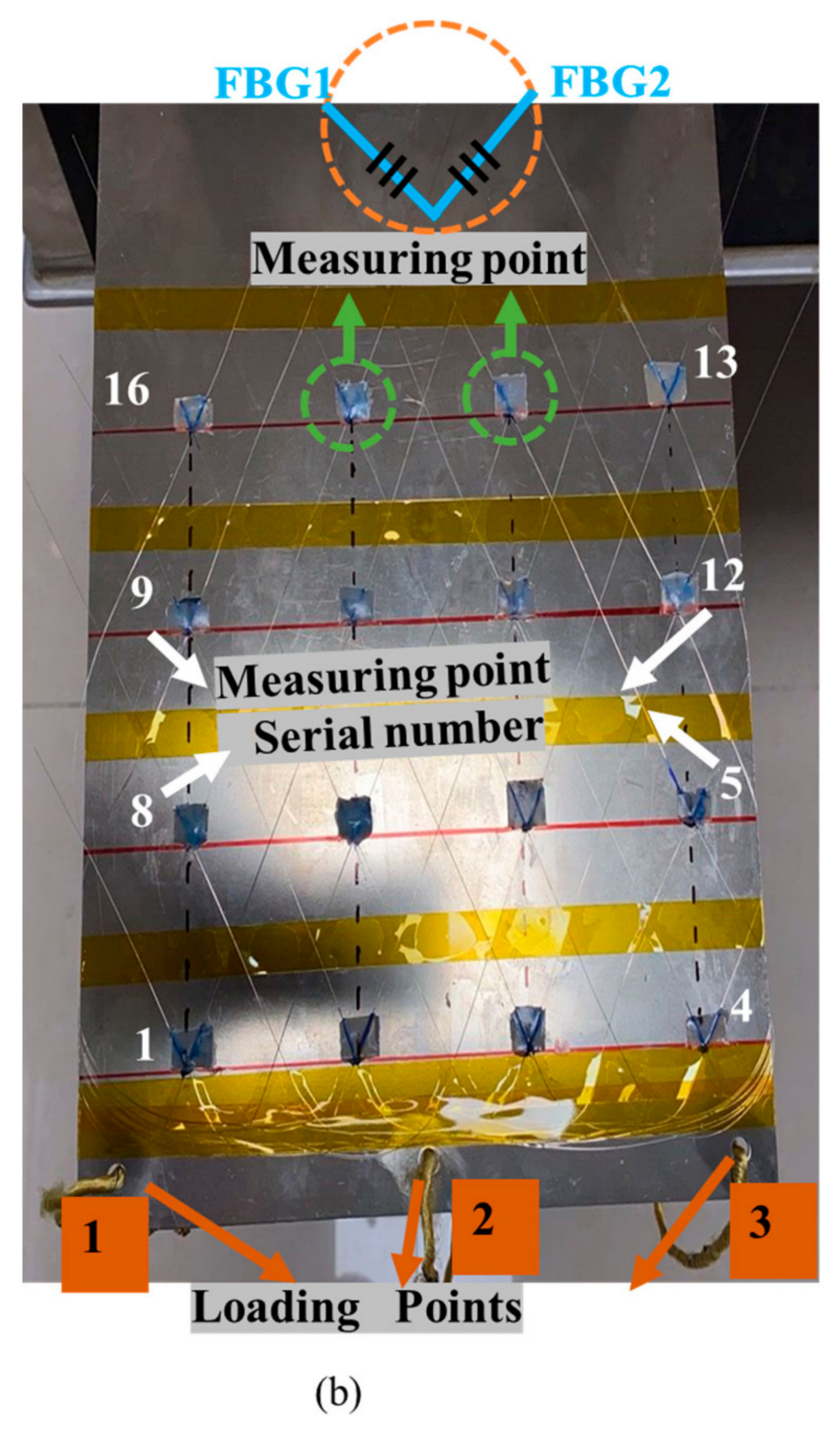
Experimental platform for structure deformation perception and shape reconstruction: (**a**) test platform; (**b**) sensor layout diagram.

The authors have verified the proposed method is feasible in theory, that of deformation sensing and shape reconstruction, and copy set up a simulation system of an experimental platform for test validation, as shown in Figure 12.

The system shown in Figure 12 consists of a computer, a four-channel high-speed wavelength demodulator, a stainless-steel thin-walled test piece, and FBGs (when FBG is attached to the test piece, each FBG is prestretched by 1.5 nm in measuring points 1–8, and by 0.8 nm in measuring points 9–16). The test piece will be deformed when it is the loaded load. First, the center wavelength drift of FBG at each measuring point can be obtained after demodulating by the high-speed wavelength demodulator. Then the strain value of the measuring point can be calculated by the central wavelength and strain conversion formula of optical fiber and the predicted values of the deformation variables of each measuring point can be obtained by the neural network model. Finally, the shape reconstruction of the test piece is completed by the interpolation method in the Matlab software version 2021.

**Figure 13:**
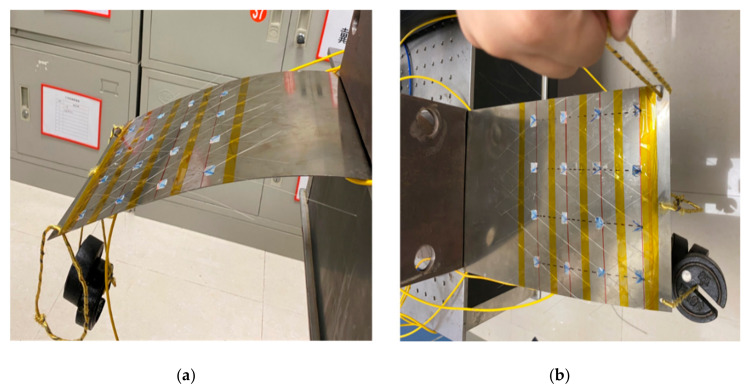
Loading mode of test piece: (**a**) Loading mode 1; (**b**) Loading mode 2.

The test piece has two types of loading, as shown in Figure 13a, a mass block is hung in the middle at loading point two of the test piece, and the weight of the mass block is 500 g, 1000 g, and 1500 g, in turn. In Figure 13b, a mass is suspended at loading point one (the weight of the mass is 500 g, 1000 g, and 1500 g, in turn), and the loading point three is forcibly lifted, as shown. Limited by experimental conditions, the deformation variables of the measuring points are measured by a laser rangefinder (the measurement accuracy is 1 mm). 

**Figure 14:**
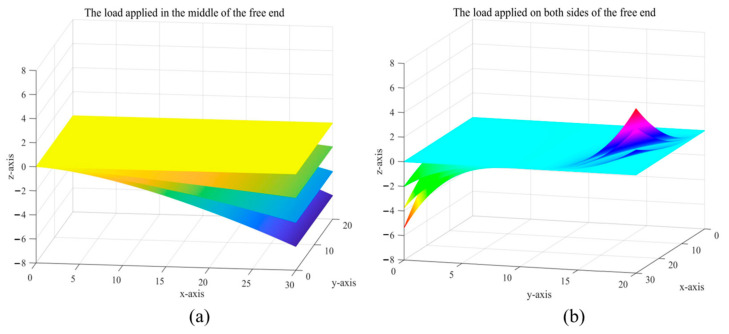
Shape reconstruction of flexible thin-walled structure: (**a**) Shape reconstruction of loading mode 1; (**b**) Shape reconstruction of loading mode 2.

In two test cases, the reconstructed shape of the test piece is shown in Figure 14.

Measuring point one and measuring point five on the test piece are selected as reference points, to compare the difference between the predicted value and the measured value of the coordinate axis. (Define the axes: y-axis—the fixed end of the flexible thin-walled structure, x-axis—side near the measuring points 1, 8, 9, and 16 of the flexible thin-walled structure)

The deformation in the y-axis direction of each measuring point on the test piece is small, and the change in the coordinate value of this direction can be ignored.

The experimental environment and equipment conditions are relatively simple, all the measured values are measured by laser rangefinder (the measurement accuracy is 1 mm). As can be seen from Table 5 and Table 6, the larger the deformation of each measuring point, the higher the reconstruction accuracy. The deformation occurs when the test piece is loaded, and in the three coordinate axes the deformation in the z-axis is large, so the reconstruction accuracy of the coordinate values of each measuring point in the z-axis is high, and the maximum reconstruction error is 15.52%, the minimum error of coordinate reconstruction of the measuring point: the y-axis is 6.11%, the x-axis is 0.22%.

The authors find that the reconstruction error is related to the selection of the coordinate origin and redefining the axis origin, as shown in Figure 1. The reconstruction errors of coordinate values of the measuring points have significant differences. It can be found that:

(1) The reconstructed error of the coordinates of the measuring points in the direction of the z-axis does not change.

The reconstruction error of the z-axis will never change if the XY coordinate axis is chosen on the thin-walled plane since the coordinate of the z-axis is perpendicular to the XY plane. The reconfiguration error of the measuring points in the z-axis direction is the measurement error of the deformation of the measuring points.

The thin walled has a larger deformation in the z-axis direction, which has a relatively large impact on shape reconstruction;

(2) The reconstruction error of the measuring points in the x y-axis direction is very much related to the selection of the coordinate origin, which is a relative error, and is not consistent with the measurement error of the form variable of the measuring points in the XY direction. Further, the deformation of the measurement points in the x- and y-axis direction is small and has limited impact on the shape reconstruction of the thin plate, so the relative error in the x- and y-axis direction can be used to verify whether the accuracy of the upgraded reconstruction method is improved.

The reconstruction error of the coordinate values of each measuring point is significantly improved compared with the previous article, and the error contrast values, as shown in the following Table 7, with the same coordinate origin.

As shown in Table 7, the error of the reconstructed values of measurement points on each coordinate axis has changed with the improved method, i.e., the maximum value of error was reduced, and the minimum value of error decreased.

## 5. Discussion

The proposed method for deformation perception and shape reconstruction of thin-walled structures is based on the data-driven mode, and similar literature is not common for shape-reconstruction applications of structures.

The error of the structure shape reconstruction proposed comes from two aspects, namely the prediction error of the model and the computational error by the interpolation method. The model prediction residuals shown in Figure 11, the maximum residuals of the model in the z-axis direction are <0.04 m, and the maximum residuals of the model in the x- and y-axis directions are <2.5 × 10^−8^ m. The influences on the subsequent shape reconstruction are all within the controllable range. In Table 5 and Table 6, the error characteristics between the measured value and the reconstructed value of the measuring point coordinates are as follows: in the z-axis, the deformation variable is the coordinate value of the measuring points, and it has a maximum error percentage of 15.52%, which contains the error of prediction and error of interpolation calculation, and the larger the deformation, the smaller the error, and the closer the reconstructed structure shape is to the actual shape. In the x-axis and y-axis, the smaller the change of the deformation variable of the measuring point, the smaller the change of its coordinate value, so the error of the coordinate value is small. The maximum coordinate error is for the x-axis 2.01% and for the y-axis 29.49%. From the reconstruction error of the coordinate value, the reconstructed shape is close to the actual shape. It shows that the method proposed in this paper is feasible and reliable.

In summary, the advantages of the reconstruction method proposed in this paper are as follows: (one) a new idea is put forward for the real-time monitoring method of thin-walled structure deformation; that is, the layout method of the sensor. The orthogonal sensor network proposed in the literature [18] is used to measure the deformation of flexible structures, however, this does not be provided a specific basis for layout. In this paper, utilizing the finite element simulation, using specific numerical analysis, the layout method of the deformation measuring sensors is quantitatively analyzed, which has a similar effect on the measurement of bending moment and torque, compared with the “M” shape sensor layout method adopted in the video of real-time strain measurement of wings released by NASA. (Two) A new mode of thinking is proposed for the deformation prediction of structure, by taking advantage of big data and establishing sample relationships between the inputs and outputs of predictive models. It only needs to ensure the accuracy and diversity of data samples, finds a suitable mapping relationship by machine learning methods and minimizes the influence of structure on deformation prediction. For example, the literature [21] also puts forward a new method for monitoring of deformation of the wing; by the von Karman strain–displacement relation, the vertical displacement and deflection angle of the morphing wing are obtained, though it only shows that the vertical displacement has a good consistency, and the nonvertical displacement is not explained.

All the experiments are conducted based on the test piece—thin-walled stainless steel. All the data in the theoretical simulation and the experimental verification have verified the feasibility and reliability of the proposed method, though the reconstruction accuracy of the method still needs to be improved. In the follow-up, further improvement to the reconstruction algorithm is needed to improve the accuracy of the whole structure shape reconstruction.

## 6. Conclusions

In this paper, Leveraging FBG in combination with data-driven models were used to establish a prediction model for structure measuring-points deformation, obtaining each point-deformation variable in the x, y, and z axes, getting the coordinate values of each measuring point on the structure after deformation, and then to complete the structure shape reconstruction by an interpolation method. The authors chose flexible thin-walled stainless steel as the test piece to establish the prediction model between the strain value and the deformation variables of the structure measuring points. The results showed that the reconstructed errors of the coordinates of the measuring points were within the acceptable range, and the reconstructed results were reliable. It provides a new method for real-time monitoring and shape reconstruction of flexible thin-walled structures such as wings, helicopter blades, wind-turbine blades, and solar panels with a high-accuracy reconstruction.

## Figures and Tables

**Table 1:** Load Information Sheet.

	Fx (mm)	Fy (mm)	Force (N)	Tx (mm)	Ty (mm)	Torque (N × mm)
Load 1	0	100	300	1	0	50,000
Load 2	66.9	44.6	−275.964	1.486	2.229	10,000
Load 3	67.11	44.74	40.58549	1.503333	2.255	225.5
Load 4	279.48	186.32	425.8678	6.210667	9.316	931.6
Load 5	0	100	300	0	0	0
Load 6	0	0	0	0	0	40,000

**Table 2:** Distribution of double FBG pinch angles.

	Angle Combination 1	Angle Combination 2	Angle Combination 3	Angle Combination 4	Angle Combination 5	Angle Combination 6	Angle Combination 7	Angle Combination 8	Angle Combination 9	Angle Combination 10
$\alpha_{1}$	0	10	20	30	40	15	30	45	60	80
$\alpha_{2}$	90	−80	−70	−60	−50	−15	−30	−45	−60	−80

**Table 3:** Information table of loads.

	x (mm)	y (mm)	Numerical Value (N)
Force	104	156	−1500.7 (N)
Torque	0	0	−728.77 (N mm)

**Table 4:** Comparison of predicted and theoretical deformation variables.

		Measuring Point 2	Measuring Point 5	Measuring Point 9	Measuring Point 16
x (m)	Theoretical value	5.43 × 10^−6^	5.50 × 10^−6^	5.50 × 10^−6^	5.19 × 10^−6^
	Predicted value	3.52 × 10^−6^	3.49 × 10^−6^	3.42 × 10^−6^	3.41 × 10^−6^
	Percentage of error (%)	35.2	36.5	37.8	34.3
y (m)	Theoretical value	6.72 × 10^−8^	1.41 × 10^−7^	3.16 × 10^−7^	6.47 × 10^−7^
	Predicted value	3.81 × 10^−8^	2.01 × 10^−7^	2.20 × 10^−7^	6.71 × 10^−7^
	Percentage of error (%)	43.3	42.6	30.3	3.7
z (m)	Theoretical value	0.1195	0.0946	0.0699	0.0442
	Predicted value	0.1123	0.0861	0.0638	0.0388
	Percentage of error (%)	6.03	8.98	9.29	12.22

**Table 5:** Figure 14a comparison table of coordinate values of measuring points.

Measuring Point	State	Axis	Reconstruction Value (cm)	Measured Value (cm)	Error (%)
measuring point 1	State I	x-axis	26.939	26.999	0.22
		z-axis	−1.83	−1.65	10.9
	State II	x-axis	26.759	26.998	0.88
		z-axis	−3.76	−3.48	8.05
	State III	x-axis	26.455	26.99	1.98
		z-axis	−5.7	−5.37	6.15
measuring point 5	State I	x-axis	21.951	21.999	0.21
		z-axis	−1.471	−1.729	14.92
	State II	x-axis	21.804	21.999	0.89
		z-axis	−2.93	−2.678	9.41
	State III	x-axis	21.556	21.998	2.01
		z-axis	−4.392	−4.107	6.94

**Table 6:** Figure 14b comparison table of coordinate values of measuring points.

Measuring Point	State	Axis	Reconstruction Value (cm)	Measured Value (cm)	Error (%)
measuring point 1	State IV	x-axis	26.895	26.789	0.4
		y-axis	0.502	0.712	29.49
		z-axis	1.269	1.477	14.08
	State V	x-axis	26.745	26.465	1.06
		y-axis	0.852	0.974	12.53
		z-axis	2.661	2.897	8.15
	State VI	x-axis	26.595	26.228	1.4
		y-axis	0.952	1.014	6.11
		z-axis	3.997	4.059	1.5
measuring point 5	State IV	x-axis	21.937	21.737	0.92
		y-axis	19.799	19.574	1.15
		z-axis	−0.936	−1.108	15.52
	State V	x-axis	21.736	21.423	1.46
		y-axis	19.599	19.418	0.93
		z-axis	−1.524	−1.769	13.85
	State VI	x-axis	21.586	21.286	1.41
		y-axis	19.198	18.898	1.59
		z-axis	−2.45	−2.67	8.24

**Table 7:** Comparison of reconstruction error values before and after method improvement.

	X (%)		Y (%)		Z (%)	
	Max	Min	Max	Min	Max	Min
Error after method improvement	2.01	0.22	29.49	1.15	15.52	1.50
Error prior method improvement	2.33	0.8	35.59	9.46	16.21	1.62

## Data Availability

Not applicable.
